# Afforestation Enhances Soil Ecosystem Multifunctionality by Improving Soil Quality and Enzyme Activities in Coastal Saline–Alkali Land

**DOI:** 10.3390/biology14111588

**Published:** 2025-11-13

**Authors:** Jianni Sun, Jiayi Yang, Xiaoyi Wang, Haifei Lu, Tailin Zhong, Haidong Xu

**Affiliations:** 1 Shandong Key Laboratory of Eco-Environmental Science for the Yellow River Delta, Shandong University of Aeronautics, Binzhou 256603, China; 2 Department of Landscape Architecture, Zhejiang Shuren University, Hangzhou 310015, China

**Keywords:** enzymatic stoichiometry, microbial nutrient limitation, nutrient cycling, soil salinity, stand ages, Yellow River Delta

## Abstract

Afforestation is critical for enhancing soil quality and optimizing land use in coastal saline–alkali regions. However, the dynamics of soil quality and ecosystem multifunctionality (EMF) during afforestation, as well as their coupling relationships, remain poorly understood. In this study, the effects of four stand ages (6, 12, 22, and 36 years) of a leguminous tree species (*Robinia pseudoacacia*) on the soil quality index (SQI), enzymatic stoichiometry, and soil ecosystem multifunctionality (EMF) were investigated in coastal saline–alkaline lands of the Yellow River Delta, China. Results showed that compared to non-afforestation, afforestation increased the SQI by 81%, 74%, 146%, and 184% in the 6-, 12-, 22-, and 36-year-old stands, respectively. Concurrently, afforestation increased the activities of the carbon acquisition (C_acq_), nitrogen acquisition (N_acq_), and phosphorus acquisition (P_acq_) enzymes and alleviated microbial N limitation. Afforestation increased the soil EMF by 182%, 243%, 263%, and 295% in the 6-, 12-, 22-, and 36-year-old stands, respectively. The SQI was significantly positively correlated with soil EMF, regardless of afforestation age. Soil salinity, SOC, TN, and TP were the best predictors of soil EMF. The afforestation of *Robinia pseudoacacia* improved the soil quality and stimulated enzyme activity, thereby increasing the soil EMF. Our findings provide a theoretical foundation for the sustainable management of stands in coastal saline–alkali lands.

## Simple Summary

Afforestation is widely recognized as an effective strategy for enhancing soil properties and ecosystem functions in coastal saline–alkali regions. However, the dynamic relationship between soil quality and ecosystem multifunctionality, as well as the underlying mechanisms driving this relationship, remains poorly understood. To fill this knowledge gap, we investigated four stand ages (6, 12, 22, and 36 years) of *Robinia pseudoacacia* in the Yellow River Delta, a representative coastal ecosystem in China. Our findings indicate that afforestation significantly enhanced soil quality compared to non-afforested sites. Afforestation also stimulated the activities of carbon-, nitrogen-, phosphorus-acquiring enzymes, and alleviated microbial N limitation with stand age. Additionally, EMF showed significant improvement across the four stand ages. Notably, we found a strong and positive correlation between SQI and EMF at all stand ages, with soil salinity and nutrients emerging as the most significant predictors of EMF. These results provide useful guidance and scientific support for improving forest management and ecological restoration efforts in this region.

## 1. Introduction

The Yellow River Delta is a fast-growing region, and its ecosystem is highly vulnerable. Soil salinization is particularly prominent in the region, with over 60% of the land affected by different degrees of salinization. The resulting chain of ecological consequences, such as soil quality degradation, biodiversity decline, and soil carbon sink reduction, seriously threatens ecosystem multifunctionality (EMF) [1,2]. Consequently, improving saline soil, enhancing soil quality, and achieving sustainable use are key to constructing an eco-economic zone in the Yellow River Delta. Phytoremediation can improve soil quality and significantly affect aboveground and belowground ecosystems and their ecological benefits [3]. Afforestation is a key pathway for rehabilitating saline–alkali lands in the Yellow River Delta. Afforestation effectively decreases salinity and pH levels in the soil while increasing soil fertility [4,5]. Additionally, afforestation affects microorganisms by altering microenvironmental conditions and soil properties. Microorganisms are highly sensitive to environmental changes, and their responses can influence multiple ecosystem functions [6,7]. However, the biological mechanisms by which afforestation affects soil quality and EMF in coastal saline–alkali lands in the Yellow River Delta remain unclear.

Soil quality is the comprehensive capacity of soil to maintain biological productivity, improve animal and plant health, and preserve environmental quality [8]. Soil quality is assessed using the soil quality index (SQI), which is calculated based on indicators of soil physical, chemical, and biochemical characteristics [9]. Soil EMF is generally used to evaluate complex and interacting biological, geochemical, and physical processes [10,11]. EMF is associated with soil nutrient cycling and the soil enzyme activities that catalyze these cycling reactions [12]. Specifically, soil enzymes promote organic matter turnover and activate unstable substances, thereby facilitating energy flow and nutrient cycling. Additionally, soil enzymes strongly influence cell metabolism, which can modulate microbial community composition by modulating resource availability for microbial growth and energy production [13,14]. Additionally, soil enzymatic stoichiometry is used to determine the relationship between microbial nutrient demands and the soil nutrient supply, as well as to evaluate microbial resource limitations, particularly for carbon (C), nitrogen (N), and phosphorus (P) [15]. Soil properties probably influence the apparent affinity and catalytic performance of element cycling-related enzymes, thereby influencing the soil EMF [16,17]. Long-term afforestation can enhance soil quality by increasing aboveground litter input, belowground root biomass, and root secretions, leading to a general increase in soil organic C, total N contents with stand age [18,19,20]. Afforestation is speculated to improve soil quality, thereby promoting microbial nutrient cycling. The changes in enzymatic stoichiometry induced by afforestation alter microbial nutrient demands, inhibiting or stimulating microbially mediated soil ecosystem functions [21,22]. Therefore, understanding the mechanism underlying the effect of afforestation on enzyme stoichiometry changes is crucial for determining effective stand age management measures to maintain soil quality and EMF in the coastal saline–alkali forest ecosystem of the Yellow River Delta.

*Robinia pseudoacacia* is a eurytopic pioneer tree species that alters soil properties through specific biological mechanisms. Its rapid growth promotes the development of a canopy and litter layer, which improves the soil microclimate. Furthermore, litter inputs and root activity accelerate the humification process and improve soil structure. As a legume, *Robinia pseudoacacia* forms symbiotic relationships with rhizobia, fixing atmospheric N and increasing soil N content. In addition, it accumulates osmolytes, such as proline and soluble sugars, to maintain cellular osmotic balance and mitigate stress-induced damage [23]. Owing to these traits, *Robinia pseudoacacia* has been widely used in the reclamation of various degraded lands worldwide, such as the Yellow River Delta [24,25]. However, stands generally decline as stand age increases, when certain limiting factors (e.g., persistent salinity and nutrient imbalance) may become dominant in this specific delta environment [26]. Whether stand decline affects soil quality and EMF, particularly its relationship with soil enzymatic stoichiometry, remains unclear. Therefore, *Robinia pseudoacacia* stands of different ages, along with un-afforested stands (considered as controls), were selected in the Yellow River Delta. In this study, we examined the relationship between soil quality and EMF by analyzing enzymatic stoichiometry and associated soil nutrients. We hypothesized that (1) afforestation on saline–alkali land increases litter and root inputs, accelerates organic matter humification, improves nutrient availability, and reduces soil salinity and pH, thereby enhancing the SQI with increasing stand age. (2) By improving soil physicochemical properties and nutrient conditions, afforestation stimulates soil enzyme activities and nutrient acquisition, leading to a significant increase in soil EMF. (3) Considering these mechanisms and the coastal saline–alkali context, soil salinity and pH are identified as the key limiting factors regulating and predicting soil EMF.

## 2. Materials and Methods

### 2.1. Study Site

This study was conducted in Gudao Town (37°35′ to 38°12′ N, 118°33′ to 119°20′ E), Dongying City, Shandong Province, China. It has a warm temperate continental monsoon type of climate, and the mean annual temperature, precipitation, and evaporation are 12.3 °C, 555.9 mm, and 1962.1 mm, respectively. The soil is classified as Solonchaks in the FAO system [27]. Owing to unique depositional settings and soil parent materials, most lands in this region have different degrees of salinization dominated by chloride salts (Cl^−^).

### 2.2. Experimental Design and Soil Sampling

Four *Robinia pseudoacacia* stands aged 6, 12, 22, and 36 years were selected in July 2024. These stand ages correspond to documented periods of major afforestation activities in Gudao Town. Unafforested wasteland was used as a control. Four plots (20 × 20 m) were established in *Robinia pseudoacacia* stands of different ages. Each plot was spaced at least 20 m apart to ensure spatial independence.

Soil was sampled at a depth of 0–20 cm with an auger (5 cm in diameter) from five random points per plot and blended to obtain the composite sample. The soil sample was subsequently separated into three portions and passed through a 2 mm sieve to remove all visible debris. One portion was frozen at 4 °C for measuring soil mineral N (NH_4_^+^-N and NO_3_^−^-N), dissolved organic carbon (DOC), and available phosphorus (AP) contents. The second portion was preserved at −20 °C for measuring the activities of hydrolytic soil enzymes associated with C-, N-, and P-acquisition. The third portion was air-dried to determine the soil salinity, pH, SOC, TN, and TP contents.

### 2.3. Edaphic Properties

The soil salinity was analyzed via gravimetry based on the mass of the residue following oven-drying at 105 °C. A glass electrode was used to measure the soil pH–water ratio at 1:2.5 (*v*/*w*). The SOC and TN contents were measured through combustion using an elemental analyzer (multi-EA 5000 Analytik, Jena AG, Jena, Germany). The soil TP content was analyzed using the molybdenum-blue approach following HClO_4_-H_2_SO_4_ digestion [28]. The soil DOC concentration was determined in fresh soil at a soil–ultrapure water ratio of 1:2 (*w*/*v*) before 5 min of centrifugation at 3000 rpm; the soil DOC concentration was later analyzed using a TOC analyzer (multiN/C3100, Jena AG, Jena, Germany). The soil mineral N concentration (NH_4_^+^-N, NO_3_^−^-N) was determined using 2 M KCl (soil–solution = 1:5 (*w*/*v*)) and examined using a continuous flow analyzer (Skalar SA-40, Skalar, Breda, The Netherlands). The soil AP concentration was determined through sodium bicarbonate extraction (soil–solution = 1:4 (*w*/*v*)) using the molybdenum-blue method [29].

### 2.4. Soil Enzyme Activities

The activities of four hydrolytic soil enzymes were examined by conducting high-throughput fluorometric assays using 96-well microtiter plates (Solarbio, Beijing, China) [30]. The soil slurry was obtained by homogenizing 2.75 g of soil with 91 mL of Tris-NaOH buffer (50 mM, pH = 8.0). The soil suspension (800 µL) was dispensed in the wells of a deep-well microplate. The enzymatic reaction was initiated by adding 200 µL of 200 mM fluorogenic substrate solution to each well. Following 4 h of incubation at 25 °C in the dark, the reaction mixtures were centrifuged for 3 min at 2960× *g* (4800 rpm). The clarified supernatant (250 µL) in each well was added to the respective wells of a black, flat-bottomed 96-well plate. Fluorescence was quantified using a TECAN Infinite M200 multifunctional microplate reader (Grödig, Salzburg, Austria) at excitation and emission wavelengths of 365 nm and 450 nm, respectively.

### 2.5. Data Analysis

The normality and homogeneity of variance of the data were analyzed by conducting the Shapiro–Wilk test and Levene’s test. The changes in each edaphic property (salinity, pH, SOC, TP, TN, DOC, NH_4_^+^-N, NO_3_^−^-N, and AP) induced by afforestation were indicated as Ln (Variable_afforestation_/Variable_Without afforestation_). The C-acquisition enzyme activity (C_acq_) was indicated as BG, the N-acquisition enzyme activity (N_acq_) was indicated as $\sqrt{\left(NAG\times LAP\right)}$, and the P-acquisition enzyme activity (P_acq_) was indicated as ALP [31].

Second, microbial resource limitations were evaluated based on the vector characteristics of the enzymatic stoichiometry [32]:

$$Vector\;length=\sqrt{x^{2}+y^{2}}$$

$$Vector\;angle=degress\;[{atan}^{2}\left(x,y\right)]$$

Here, *x* indicates the relative ratio of C_acq_ to P_acq_, and y indicates the relative ratio of C_acq_ to N_acq_.

For the SQI after afforestation, every edaphic indicator was transformed into a score between 0 and 1 [33]:

$$S_{Li}=X/X_{max}$$

$$S_{Li}=X_{min}/X$$

Here, *S_Li_* represents the linear score of parameters i within 0–1, and *X*, *X_max_*, and *X_min_* represent the analyzed values and the maximum and minimum means of parameter *i*, respectively. The soil indicators were classified into two groups: “more is better” (SOC, TP, TN, DOC, NH_4_^+^-N, NO_3_^−^-N, and AP) and “less is better” (salinity and pH).

Soil EMF was assessed based on the activities of carbon-cycling enzymes (β-glucosidase, BG), nitrogen-cycling enzymes (N-acetylglucosaminidase, NAG; leucine aminopeptidase, LAP), and a phosphorus-cycling enzyme (alkaline phosphatase, ALP). These enzymes participate in elemental cycling processes and were therefore used as indicators of ecosystem functions [22,34].

The EMF was quantified by normalizing soil enzyme activities with the *Z*-*score* method, which is widely employed in contemporary studies on ecosystem multifunctionality [35,36,37].

$$Z-score=(x-{mean}_{i})/{SD}_{i}$$

Here, *x* represents the analyzed enzyme activity, the mean represents the mean of enzyme *i*, and *SD* represents the standard deviation of enzyme *i*.

Third, the effects of afforestation on edaphic properties (salinity, pH, SOC, TP, TN, DOC, NH_4_^+^-N, NO_3_^−^-N, and AP), soil enzyme activities (C_acq_, N_acq_, and P_acq_), soil enzymatic stoichiometric characteristics (vector length and vector angle), SQI, and soil EMF were tested via one-way ANOVA. In the case of significant effects, we performed post hoc tests with Fisher’s least significant difference (LSD) test, with a significance level of α = 0.05. Pearson correlation analysis was performed to determine the relationships of edaphic properties with soil enzyme activity and stoichiometric characteristics. The relationships between the SQI and the soil EMF were tested by conducting regression analysis. Random forest analysis was performed to analyze the importance and statistical significance of edaphic properties on the soil EMF. All statistical analyses were performed using R Version 4.0.5.

## 3. Results

### 3.1. Influence of Afforestation on Edaphic Properties

**Figure 1:**
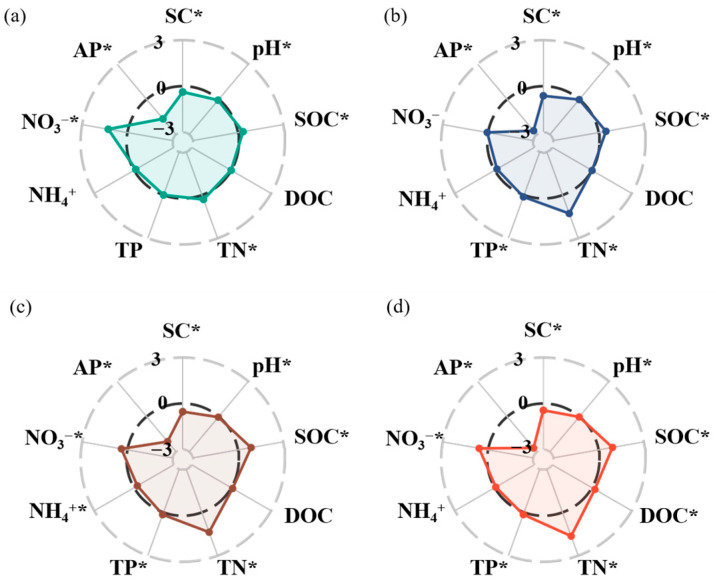
Radar graphs reflecting the responses of soil physical and chemical properties of soils to different stand ages ((**a**) 6 years; (**b**) 12 years; (**c**) 22 years; (**d**) 36 years). * indicates significant differences between soils without and with *Robinia pseudoacacia* (*p* < 0.05). An effect size > 0 indicates a positive response to stand age of *Robinia pseudoacacia*, and an effect size < 0 indicates a negative response to stand age of *Robinia pseudoacacia*.

Afforestation significantly altered nearly all edaphic properties, but the magnitude of these effects differed among the variables. The soil salinity, pH, and AP were significantly lower with afforestation than without afforestation. The soil salinity decreased by 38%, 63%, 53%, and 44% in the 6-, 12-, 22-, and 36-year-old stands, respectively (*p* < 0.05, Figure 1a–d). The soil pH decreased by 7%, 2%, 4%, and 2% in the 6-, 12-, 22-, and 36-year-old stands, respectively (*p* < 0.05, Figure 1a–d). The soil AP decreased substantially by 166%, 268%, 213%, and 267% in the 6-, 12-, 22-, and 36-year-old stands, respectively (*p* < 0.05, Figure 1a–d). However, afforestation significantly increased the SOC and TN contents. In the 6-, 12-, 22-, and 36-year-old stands, the SOC content increased by 29%, 45%, 83%, and 88%, respectively, and the TN content increased by 31%, 128%, 138%, and 165%, respectively (*p* < 0.05, Figure 1a–d). Moreover, the soil NO_3_^−^-N content increased by 127%, 40%, and 59% in the 6-, 22-, and 36-year-old stands, respectively (*p* < 0.05, Figure 1a,c,d). The soil TP content increased by 13%, 16%, and 15% in the 12-, 22-, and 36-year-old stands, respectively (*p* < 0.05, Figure 1b–d). Additionally, the soil DOC content increased by 22% in the 36-year-old stand (*p* < 0.05, Figure 1d), whereas the soil NH_4_^+^-N content increased by 25% in the 22-year-old stand (*p* < 0.05, Figure 1c).

### 3.2. Influence of Afforestation on Soil Enzyme Activities and Stoichiometric Characteristics

**Figure 2:**
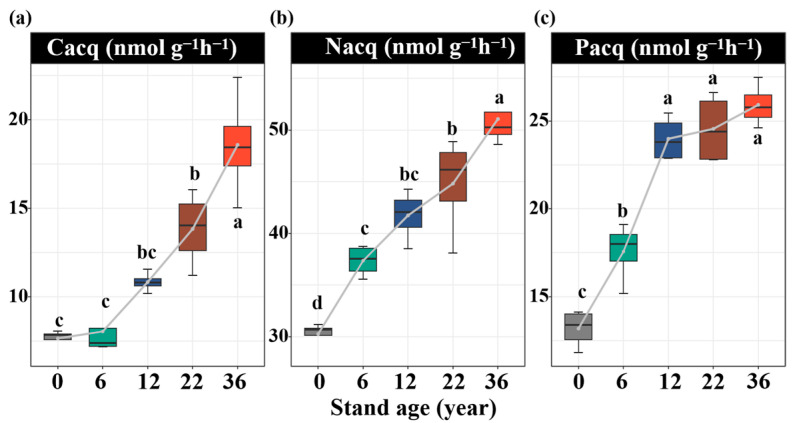
Variations in carbon acquisition (C_acq_, (**a**)), nitrogen acquisition (N_acq_, (**b**)), and phosphorus acquisition (P_acq_, (**c**)) enzyme activities with stand ages. Values are shown as means ± standard error (n = 4). Bars with different lowercase letters are significantly different (*p* < 0.05) based on Tukey’s HSD tests.

**Figure 3:**
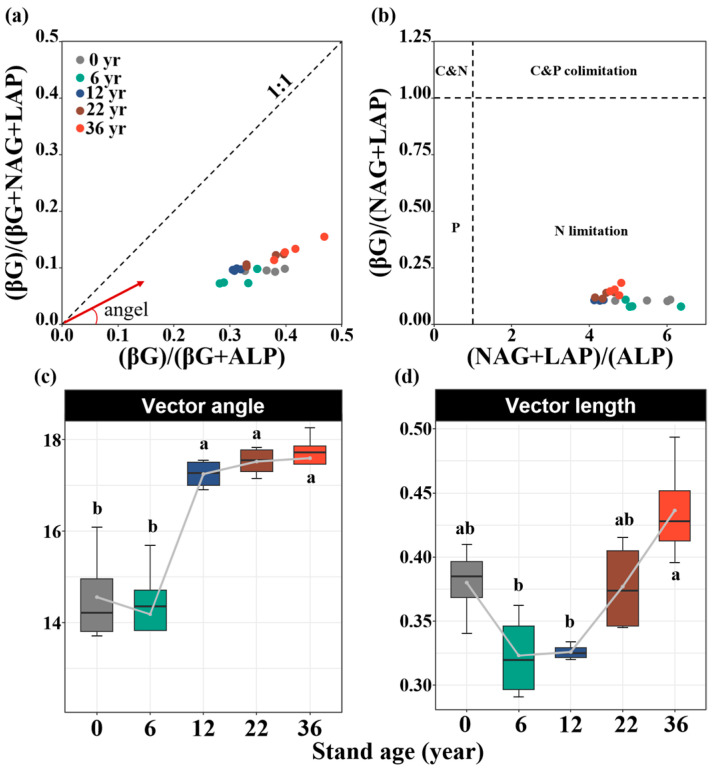
Extracellular enzymatic stoichiometry of the relative proportions of C_acq_ to N_acq_ versus C_acq_ to P_acq_ in response to different stand ages (**a**). Stoichiometric analysis of enzyme activities to identify the C, N, and P limitations for microorganisms in soil (**b**). Using 1.0 as a horizontal and vertical baseline along the axes of enzyme activity ratios ((NAG + LAP)/ALP on the x-axis and (BG)/(NAG + LAP) on the y-axis), four groups of microbial resource limitations were identified: N limitation, P limitation, C and N co-limitation, and C and P co-limitation. Vector angle (**c**) and vector length (**d**) in response to different stand ages. Values are shown as means ± standard error (n = 4). Bars with different lowercase letters are significantly different (*p* < 0.05) based on Tukey’s HSD tests.

**Figure 4:**
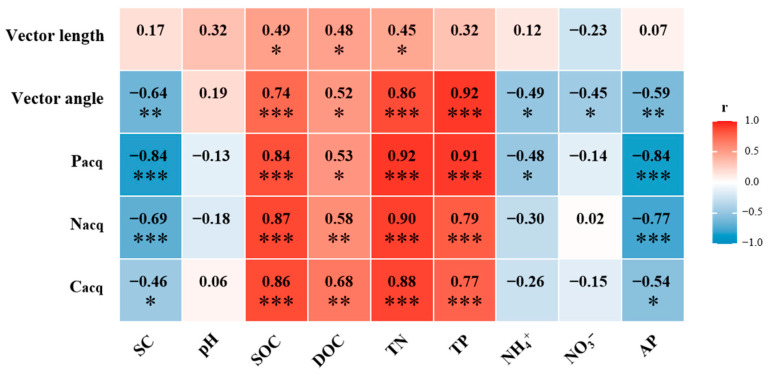
Relationships between vector length, vector angle, C_acq_, N_acq_, and P_acq_ enzyme activities and edaphic properties. Significance levels are indicated as follows: * *p* < 0.05, ** *p* < 0.01, *** *p* < 0.001.

Soil enzyme activities were significantly influenced by afforestation. Compared to that without afforestation, the soil C_acq_ enzyme activity increased by 35%, 58%, and 88% in the 12-, 22-, and 36-year-old stands, respectively (*p* < 0.05, Figure 2a). The soil N_acq_ enzyme activity increased by 21%, 32%, 39%, and 52% in the 6-, 12-, 22-, and 36-year-old stands, respectively (*p* < 0.05, Figure 2b). The soil P_acq_ enzyme activity increased by 29%, 60%, 62%, and 68% in the 6-, 12-, 22-, and 36-year-old stands, respectively (*p* < 0.05, Figure 2c). All results of the soil enzyme stoichiometry vectors were under the dashed line (1:1 line), suggesting the strong N limitation of the soil microbial community (Figure 3a,b). The vector angle increased with stand age, which intensified N limitation (Figure 3c). The vector length initially decreased but then increased, which induced an increase in C limitation (Figure 3d). The activities of the soil C_acq_, N_acq_, and P_acq_ enzymes were significantly negatively related to the soil salinity and AP content and significantly positively related to the SOC, DOC, TN, and TP contents (Figure 4). The vector angle was significantly positively related to the soil salinity, NH_4_^+^-N, NO_3_^−^-N, and AP contents but significantly negatively related to the SOC, DOC, TN, and TP contents (Figure 4). The vector length was significantly positively related to the SOC, DOC, and TN contents (Figure 4).

### 3.3. Influence of Afforestation on SQI and EMF

**Figure 5:**
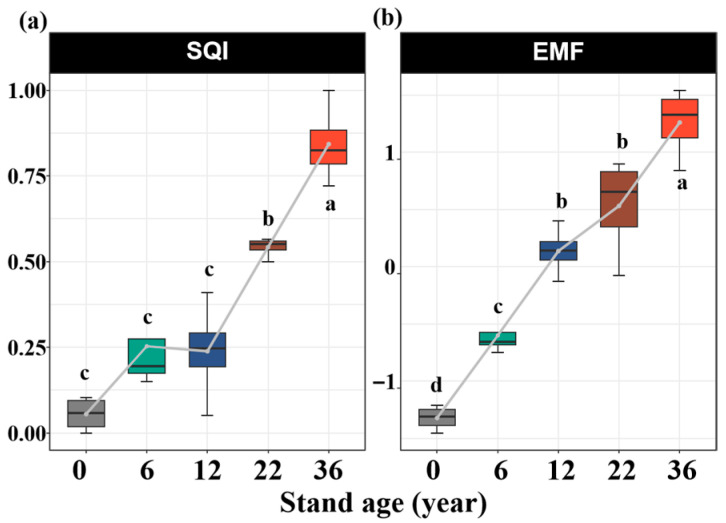
Variations in soil quality index (**a**) and ecosystem multifunctionality (**b**) with stand ages. Values are means ± standard error (n = 4). Bars with different lowercase letters are significantly different (*p* < 0.05) based on Tukey’s HSD tests.

**Figure 6:**
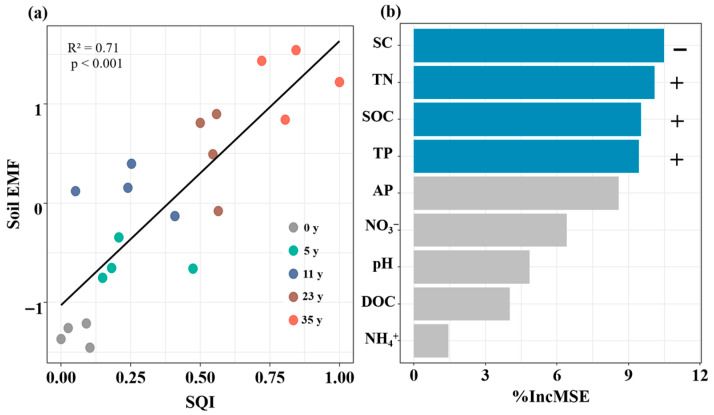
Relationship between soil ecosystem multifunctionality and soil quality index via line regression analysis (**a**). Random Forest mean predictor importance (% increase in MSE) of edaphic properties on soil EMF (**b**). Blue bars indicate significant relationships, gray bars indicate insignificant relationships. + indicates positive relationships, − indicates negative relationships.

Afforestation affected the SQI and EMF. Compared to that without afforestation, the SQI increased by 81%, 74%, 146%, and 184% in the 6-, 12-, 22-, and 36-year-old stands, respectively (Figure 5a). The soil EMF increased by 182%, 243%, 263%, and 295% in the 6-, 12-, 22-, and 36-year-old stands, respectively (Figure 5b). The soil EMF was strongly correlated with the SQI (Figure 6a). Based on random forest model analysis, the contributions of edaphic properties accounted for 85% of the soil EMF variability (Figure 6b). Soil salinity, TN, SOC, and TP were the key factors driving the soil EMF (Figure 6b).

## 4. Discussion

### 4.1. Effect of Afforestation on Edaphic Properties with Afforestation Age

These findings support our first hypothesis that soil salinity and pH decrease significantly with increasing stand age (Figure 1). This phenomenon stems from a series of interconnected physical, biological, and biogeochemical processes initiated by afforestation. First, afforestation increases ground cover, reducing surface evaporation and consequently decreasing salt accumulation in the topsoil [38,39]. As stand age increased, the canopy density of *Robinia pseudoacacia* and the thickness of the surface litter layer increased, which increased the water-retention capacity of the soil, creating favorable conditions for salt leaching [4]. Second, afforestation markedly improved the soil physical structure, as indicated by an increase in the proportion of macroaggregates [40]. This structural change could reduce surface runoff and increase water infiltration. More importantly, the production of humic acids and other organic acids was increased during litter decomposition, altering the soil chemical environment [41]. The H^+^ produced during litter decomposition exchangeable basic cations (e.g., Ca^2+^, Mg^2+^, and Na^+^) from soil colloids, allowing them to enter the soil. Meanwhile, improvements in soil physical structure and infiltration capacity are an effective way for displaced cations and their associated anions (e.g., Cl^−^ and SO_4_^2−^) to be leached out of the soil [42]. Acidification makes basic ions mobile, while enhanced infiltration and leaching remove them; the two work synergistically to effectively transform the soil from a slightly alkaline to a slightly acidic state [43]. Additionally, the SOC and TN contents increased significantly with stand age, whereas the AP content decreased significantly (Figure 1). Litter inputs and root-derived carbon are the primary sources of SOC accumulation in forests. The aboveground productivity of *Robinia pseudoacacia* increased with stand age, leading to greater litter input, which directly contributed to the accumulation of SOC and TN [44]. Concurrently, root turnover and root exudates contributed substantial amounts of labile carbon compounds to the soil, further contributing to the accumulation of SOC and TN [45]. Furthermore, as a legume, *Robinia pseudoacacia* efficiently fixes atmospheric N through symbiotic rhizobia [46]. Both root biomass and N-fixing capacity increased with stand age, providing a significant supplementary source of soil N. In contrast, the AP content decreased with stand age, likely due to increased P uptake by both the *Robinia pseudoacacia* and soil microbial biomass, which likely gradually offset and exceeded P inputs derived from litter decomposition and root exudates [47,48]. In addition, the soil acidification may enhance the chemical fixation of P, further reducing the soil AP pool. Our findings indicate that *Robinia pseudoacacia* afforestation effectively enhances soil quality, with this positive effect becoming more pronounced as stand age.

### 4.2. Effect of Afforestation on Soil Enzyme Activity and Stoichiometry with Afforestation Age

Afforestation significantly increases the activities of the C_acq_, N_acq_, and P_acq_ enzymes, which are driven primarily by the evolution of edaphic properties with stand age. Soil enzyme activity is largely regulated by changes in soil nutrient availability [49]. An increase in litter input elevated the amount of soil microbial biomass carbon and stimulated microbial metabolic activity [50]. Soil microorganisms accelerate the mineralization and utilization of nutrients (N and P) by increasing the production of corresponding hydrolases and oxidases to assimilate and use these nutrients from litter for growth [51]. However, our findings revealed that despite an increase in litter-derived C inputs, the vector length increased, indicating that microbial C limitation intensified with stand age (Figure 3d), which may be explained in three ways. First, while increased litter input stimulates enzyme production, the synthesis of enzymes demands substantial amounts of carbon and energy [52]. Second, the accumulation of recalcitrant compounds in litter may require more specialized hydrolases and oxidases for decomposition, further increasing C resource demand [53]. Finally, the litter and root exudates of *Robinia pseudoacacia* plants contain relatively high N contents, and microorganisms consume additional C and energy to metabolize and utilize this N [44]. Consequently, the afforestation of *Robinia pseudoacacia* stimulated microbial metabolism, combined with the microbial demand for N and P, which contributed to an increase in enzyme activity. The vector length was positively associated with the NH_4_^+^-N, NO_3_^−^-N, and AP contents, suggesting that a relatively high soil N content was associated with greater microbial C limitation, reflecting an increase in C consumption to meet N metabolic demands [54]. Moreover, the vector angles were smaller than 45° at all stand ages, indicating microbial N limitation in coastal saline–alkali land (Figure 3a), which is consistent with findings in temperate forest ecosystems [55]. This persistent N limitation aligns with the consistently high activities of NAG and LAP enzymes observed in our study, demonstrating a microbial strategy to increase N_acq_ enzyme production in response to N scarcity. The vector angle was negatively related to soil salinity but positively related to C and nutrient availability, suggesting that the afforestation of *Robinia pseudoacacia* can alleviate microbial N limitation by reducing soil salinity and increasing the SOC, N, and P contents with stand age (Figure 4). Therefore, the afforestation of *Robinia pseudoacacia* effectively increases the activity of soil enzymes driving key biogeochemical cycles, potentially contributing significantly to the improvement in soil EMF, which supports our second hypothesis.

### 4.3. Main Factors Affecting EMF with Afforestation Age

In contrast to our third hypothesis, soil salinity, TN, SOC, and TP, rather than soil pH, are key predictors of soil EMF. In this study, the activities of the C_acq_, N_acq_, and P_acq_ enzymes increased significantly with stand age, likely driven by a decrease in soil salinity and an increase in nutrient availability. These changes collectively stimulate microbial activity and enzyme production, ultimately increasing the soil EMF [2]. Random forest analysis confirmed that an increase in SQI enhances enzyme activities, supporting key biogeochemical processes and thereby promoting soil EMF. Additionally, microenvironments with a greater soil quality probably activate microorganisms, increasing their diversity and richness, thereby promoting the formation of microbial hotspots related to nutrient cycling and soil organic matter decomposition, ultimately synergistically enhancing EMF [35]. This was supported by the positive relationship between the SQI and the soil EMF (Figure 6a). Thus, we speculated that during the phytoremediation of coastal saline–alkali land, the progressive improvement in soil quality with stand age not only effectively activates key ecosystem functions (such as nutrient cycling and organic matter decomposition) but also synergistically promotes multiple ecosystem functions.

### 4.4. Limitations and Outlook

In this study, we used a space-for-time substitution approach to analyze the relationship between soil quality and EMF across different stand ages, with a focus on soil microbial enzyme stoichiometry and associated soil nutrient dynamics. However, the relationship was limited, and the limited range of stand ages may have influenced our findings. Therefore, large-scale studies with more data are needed to validate and expand our findings. First, long-term studies that cover broader stand age gradients are needed to comprehensively understand the relationship of the SQI with the soil EMF and identify the inflection points in the SQI and the key ecological thresholds [56,57]. Second, soil enzyme activities are regulated not only by resource inputs and nutrient limitations but also closely by structural and functional shifts in soil microorganisms. We did not investigate the successional processes of soil microorganisms with stand age or their linkages to enzyme stoichiometry and EMF [58]. The application of high-throughput sequencing and functional gene quantification techniques can elucidate the mechanisms linking the abundance and diversity of specific functional microbial groups (e.g., P-solubilizing bacteria, N-fixing bacteria, and C-degrading bacteria) with enzyme activity, nutrient cycling, and EMF. Finally, microorganisms in deep soil are significantly distinct and may differ fundamentally from those in topsoil, which serves as the primary microbial habitat [59]. Future research should investigate the relationship between microbial diversity and soil EMF in deep soil to provide a scientific basis for comprehensively assessing the ecological functions of plantation soils and developing ecological restoration strategies based on stand age.

## 5. Conclusions

The afforestation of *Robinia pseudoacacia* initiated key pedogenic and biological processes that enhanced soil quality and ecosystem multifunctionality in coastal saline–alkali soils. As the stand ages, afforestation promoted humification and soil structure improvement by increasing litter input and enhancing root–soil interactions, which in turn facilitated salt leaching and led to reduced soil salinity and pH. Concurrently, afforestation stimulated microbial activity, which was reflected in increased enzyme activity and an alleviation of microbial N limitation. The decomposition and nutrient mineralization mediated by enzymes could strengthen soil EMF, with reduction in salinity and increase in soil nutrients. The management strategy of combining optimal stand age with N fertilization could be a viable approach for improving soil quality and ecosystem multifunctionality in coastal saline–alkaline lands.

## Data Availability

The original contributions presented in the study are included in the article. Further inquiries can be directed to the corresponding author.
